# Prevalence of self-reported multimorbidity in the general population and in primary care practices: a cross-sectional study

**DOI:** 10.1186/s13104-016-2121-4

**Published:** 2016-06-17

**Authors:** Nadjib-Mohamed Mokraoui, Jeannie Haggerty, José Almirall, Martin Fortin

**Affiliations:** Faculty of Medicine and Health Sciences, Université de Sherbrooke, Sherbrooke, QC Canada; Faculty of Medicine, McGill University, Montreal, Canada; Département de médecine de famille et de médecine d’urgence, Université de Sherbrooke, 2500 boul. de l’Université, Sherbrooke, QC J1K 2R1 Canada; Unité de médecine de famille, 305 St-Vallier, Chicoutimi, PQ G7H 5H6 Canada

**Keywords:** Multimorbidity, Prevalence, Primary care, General population

## Abstract

**Background:**

Settings affect estimation of multimorbidity prevalence. Multimorbidity prevalence was reported to be substantially higher among family practice-based patients than in the general population, but prevalence estimates were obtained with different methods and at different time periods. The aim of the present study was to compare estimates of the prevalence of multimorbidity in the general population and in primary care clinical practices, both measured simultaneously and with the same methods.

**Methods:**

Cross-sectional analysis of results from the Program of Research on the Evolution of a Cohort Investigating Health System Effects (PRECISE) in Quebec, Canada. Subjects aged between 25 and 75 years. A randomly-selected cohort in the general population recruited by telephone, and patients recruited in the waiting room of 12 primary care clinics. Prevalence of multimorbidity was estimated using three operational definitions of multimorbidity: (a) two or more chronic conditions (MM 2+); (b) three or more chronic conditions (MM 3+); and (c) disease burden morbidity assessment score of 10 or higher (DBMA 10+).

**Results:**

Prevalence in the general population ranged from 59.4 % (with MM2+) to 16.9 %, (with DBMA10+). In primary care practices, prevalence estimates ranged from 69.5 to 29.5 %. Prevalence estimates of multimorbidity were about 10 % higher in primary care clinical practices than in the sample from the general population. The difference was not importantly affected by the use of different operational definitions of multimorbidity. Also, there was a higher burden of disease among patients attending primary care clinics.

**Conclusions:**

The study suggests that the problem of multimorbidity in the two settings is different both quantitatively (a higher proportion of patients with multimorbidity in primary care clinical practices), and qualitatively (a higher disease burden of patients attending primary care clinics). For decision-makers interested in resource allocation, prevalence estimates in samples from primary care practices are more informative than estimates in the general population, but burden of disease should also be considered as it results in more complexity in primary care clinical practices.

## Background

Point prevalence is the proportion of a population affected by a health condition at a given moment in time and it provides a snapshot of the burden of disease in that population. Multimorbidity is the co-occurrence of chronic illnesses in the same person and its prevalence provides a picture of the complexity of the burden of chronic illness. It has been reported that settings (general population, primary care practices) affect estimation of prevalence of multimorbidity [1–3]. In a previous study we suggested that age-standardized estimates of multimorbidity prevalence were substantially higher in a family practice-based sample of patients than in the general population [2]. However, the prevalence estimates in the two populations were obtained with different methods of collecting data, and conducted at different time periods [2]. Consequently, it was not possible to conclude definitively the extent to which prevalence estimates differ in these two study populations. We also suggested that estimates are not only higher in primary practice samples when multimorbidity was defined as two-plus chronic diseases, but also that the difference in prevalence with the general population became more marked when multimorbidity was defined as three-plus chronic diseases [2]. Compared to two or more diagnoses, the latter definition results in a lower prevalence of multimorbidity and likely better identifies patients with higher needs, and thus may be more meaningful for clinicians than a count of two or more, which is less discriminating. However, additional research is needed about the effects of using different definitions of multimorbidity [4].

The principal aim of the present study was to compare estimates of the prevalence of multimorbidity in the general population and in primary care clinical practices, both measured simultaneously, in the same region and with the same methods. The secondary objective of the study aimed at exploring the effect of using different operational definitions of multimorbidity on the differences of prevalence observed between the two sampled populations.

## Methods

### Study design and setting

The present cross-sectional study is a secondary analysis conducted among parallel samples of the general population and primary care patients recruited for a larger cohort study (the Program of Research on the Evolution of a Cohort Investigating Health System Effects, PRECISE) [5] within the geographic boundaries of four local healthcare networks in Quebec, Canada. These networks are located in metropolitan, urban, rural and remote areas. The study was approved by the scientific and ethics committees of the Centre de santé et de services sociaux de Chicoutimi, as well as the Research Ethics Committee of Hôpital Charles Lemoyne, Quebec.

### Participants

All participants had to be aged between 25 and 75 years, able to respond to written and oral questions in English or French and reside in one of the four networks identified. The general population sample was selected by random digit dialing from March to April 2010 within the administrative boundaries of the four networks. Once contact was made, staff selected the eligible adult in the household with the most recent birthday to ensure random selection.

The primary care patient sample was also recruited from March to April 2010 by research assistants recruiting patients in the waiting room of 12 primary care clinics located within the networks identified. In each of the four networks, we purposefully selected three sentinel clinics typical of the dominant forms of primary healthcare organizations: private medical clinics, community health clinics, and Family Medicine Groups. To be included in the study, participants had to be a regular patient of the clinic and be consulting for themselves in addition to be aged between 25 and 75 years, able to respond to written and oral questions in English or French and reside in one of the four networks identified.

### Data collection

At recruitment, participants reported on socio-demographic information (age, gender, education level, perceived financial situation, house ownership, presence or absence of medical insurance, the possession of a retirement plan). Based on these data, we produced a data-driven classification of socio-economic status and classified patients into four socio-economic clusters: elite group, middle-high, middle-low, and low. Definitional criteria for the four socio-economic clusters have been previously described [6]. After recruitment, a self-administered questionnaire was mailed to the subjects included in the general population sample. The same questionnaire was given to the patients recruited in primary care. Among other instruments, the questionnaire included the disease burden morbidity assessment (DBMA) [7, 8]. The instrument elicits whether the patient has been diagnosed by a health professional with or is taking any medications from a list of 21 conditions [7, 8]. For each condition present, the patient assesses the degree to which the condition limits his/her daily activities on a five-point descriptive scale from 1 = “not at all” to 5 = “a lot”; absence of the conditions is scored zero. The total DBMA score is the sum of the limitation from all conditions. We also made a simple count of chronic conditions present in each subject from the list of 21 conditions.

We used three operational definitions for multimorbidity: (1) the presence of two or more chronic conditions (MM 2+); (2) the presence of three or more chronic conditions (MM 3+); (3) DBMA score of 10 or higher (DBMA 10+). The definition of multimorbidity based on DBMA 10+ arises from the clinical experience that it is a threshold of the DBMA score that may correspond to patients with several chronic diseases that individually have a minimal impact on the daily living of a patient, or the presence of at least two chronic diseases with an important impact on patient’s daily living. This definition of multimorbidity was proposed in the protocol of the project PRECISE [5], and used in that study.

### Data analysis

The sociodemographic characteristics as well as number of diseases and DBMA scores of the samples were analyzed with descriptive statistics. To compare the extent to which the two samples differed, the Student’s t test was used to measure possible differences of continuous variables, and the Chi squared test was used to test possible differences between categorical variables. Subsequently, we calculated age-standardized prevalence by direct standardization using the general population of Quebec as a Ref. [9]. To determine the extent to which the estimates where statistically significantly in the samples, we calculated Fisher’s 95 % confidence intervals (95 % CI) looking for overlap. No overlap of 95 % CI was considered a statistical significant difference. All the analyses were done using SPSS version 20. The alpha significance level was set at 0.05.

## Results

In the general population, a total of 2458 subjects were contacted. Among them, 2409 subjects were eligible, and 1718 (70 % of those initially contacted) returned the completed questionnaires. In the primary care clinics, 1058 subjects were contacted, and 1029 of them were eligible. Questionnaires were returned by 789 subjects, that is, 75 % of the subjects initially contacted.

Characteristics of participants in both samples are shown in Table 1. Age and sex distribution were significantly different between the samples from the two settings, being the sample from primary care clinics older and with a greater proportion of female subjects. The burden of disease, measured either by the number of chronic conditions or by the DBMA score was significantly higher in the whole sample from primary care clinics than in the one from the general population.

**Table 1:** Characteristics of participants

	Mean (SD)		p
	General population n = 1718	Primary care n = 789	
Age, year	51.3 (12.5)	53.0 (12.9)	0.002
Number of chronic conditions	2.6 (2.5)	3.3 (2.8)	<0.001
Chronic conditions in subgroup MM 2+	4.1 (2.1)	4.6 (2.4)	<0.001
Chronic conditions in subgroup MM 3+	4.9 (2.0)	5.3 (2.3)	0.002
DBMA score	4.9 (6.0)	6.9 (7.7)	<0.001
DBMA in subgroup DBMA 10+	15.6 (6.3)	17.2 (7.8)	0.017

	N (%)		p
Males	699 (40.7)	253 (32.1)	<0.001
Education level			
Incomplete secondary school or lower	378 (22.0)	153 (19.4)	0.097
Completed secondary school	521 (30.3)	263 (33.3)	
College	393 (22.9)	189 (24.0)	
University	418 (24.3)	164 (20.8)	
Missing data	8 (0.5)	20 (2.5)	
Socio-economic classes			
Elite group	338 (19.7)	155 (19.6)	0.317
Middle-high	737 (42.9)	335 (42.5)	
Middle-low	345 (20.1)	138 (17.5)	
Low	230 (13.4)	122 (15.5)	
Missing data	68 (4.0)	39 (4.9)	

*DBMA* disease burden morbidity assessment; *MM 2+* subjects with two or more chronic conditions; *MM 3+* subjects with three or more chronic conditions; *DBMA 10+* subjects with DBMA score of 10 or higher

Although sex distribution was different between the samples, prevalence estimations by sex within each sample were not significantly different for any of the three operational definitions of multimorbidity (see Appendix). Therefore, prevalence difference between the two cohorts was analyzed by grouping males and females. Table 2 shows prevalence estimates in the general population and primary care practices according to the different operational definitions of multimorbidity. Prevalence in the general population ranged from 59.4 %—for the operational definition that included subjects with less disease burden (MM2+)—to 16.9 %, for the definition selecting sicker subjects (DBMA10+). In primary care practices, prevalence estimates ranged from 69.5 to 29.5 %. The difference between the two settings across all definitions was about 10 % higher in primary care. To further analyze the observed differences, we estimated age-standardized prevalence to compare the two cohorts. Table 3 shows the age-standardized prevalence in the two cohorts according to the different operational definitions of multimorbidity. The age-standardized prevalence estimates were somewhat lower than those non-standardized (mean difference of 1.4 %), but there was still a difference between the two samples which varied from 8.1 to 9.3 % across the different operational definitions of multimorbidity. In all analyses, there was no overlap of 95 % confidence intervals of prevalence estimates in the two samples with any of the definitions of multimorbidity.

**Table 2:** Prevalence in the general population and primary care practices according to different operational definitions of multimorbidity

	General population prevalence (95 % confidence interval)	Primary care prevalence (95 % confidence interval)	Difference %
MM 2+	59.4 (56.8–61.8)	69.5 (66.0–72.6)	10.1
MM 3+	43.7 (41.2–46.2)	54.4 (50.6–57.8)	10.7
DBMA 10+	16.9 (15.2–18.8)	26.5 (23.2–29.4)	9.6

*MM 2+* subjects with two or more chronic conditions; *MM 3+* subjects with three or more chronic conditions; *DBMA 10+* subjects with DBMA score of 10 or higher

**Table 3:** Age-standardized prevalence in the general population and primary care practices according to different operational definitions of multimorbidity

	General population prevalence (95 % confidence interval)	Primary care prevalence (95 % confidence interval)	Difference %
MM 2+	53.68 (53.64–53.72)	62.94 (62.90–62.97)	9.3
MM 3+	37.75 (37.71–37.79)	45.85 (45.81–45.89)	8.1
DBMA 10+	14.19 (14.16–14.22)	22.94 (22.90–22.97)	8.8

*MM 2+* subjects with two or more chronic conditions; *MM 3+* subjects with three or more chronic conditions; *DBMA 10+* subjects with DBMA score of 10 or higher

## Discussion

Prevalence estimates of multimorbidity are important for reporting about the health status of a given population. Measurements of multimorbidity in different settings are expected not only to generate different estimates of prevalence, but also to provide different information [1–3]. Estimates of the prevalence of multimorbidity based on measures in a sample of the general population provide information about the magnitude of the burden of chronic illness in a given location. On the other hand, measuring the prevalence of multimorbidity in a sample of patients attending clinical consultation in primary care practices provides insight into the physician’s daily work. Looking at the results in primary care practices, we may say that almost half of patients attending primary care clinics had multimorbidity, as defined by MM3+, and almost one patient out of four had high disease burden, as defined by DBMA 10+. This way of looking at the situation probably provides a better idea of the workload faced by physicians in primary care practices.

As we mentioned before, previous studies comparing prevalence of multimorbidity in the general population and the population of patients attending clinical consultation in primary care had different methods of collecting data that could have influenced the results [1–3]. The present study used the same methods of data collection in both populations and showed that prevalence estimates of multimorbidity measured in a sample of patients from the waiting room of primary care clinics were about 10 % higher than estimates in a sample from the general population. The difference between the two samples was similar across the three operational definitions of multimorbidity used to measure the prevalence. Two important conclusions can be drawn from this. First, the difference is smaller than previously reported (roughly, 35 %) [2] but could still be explained by the oversampling of frequent attendees with higher needs in primary care practices which is an important reality. Secondly, at variance with what was previously reported, the difference remains stable across definitions of multimorbidity when the same method is used in both cohorts.

A closer look at the data showed that the increased prevalence in the primary care practices was only one aspect of the difference in multimorbidity between the two samples. Within each sample, choosing operational definitions of multimorbidity that include subjects with increased disease burden (MM2 + < MM3 + < DBMA 10+) have the effect of decreasing prevalence estimates. However, in the sample from primary care clinics we observed not only higher prevalence estimates of multimorbidity (Table 2) but also higher burden of disease (Table 1) than in the sample from the general population for each operational definition. This means that the workload generated by multimorbidity in primary care practices, as compared with the general population, includes two elements: (1) a higher proportion of patients with multimorbidity; and (2) a higher burden of disease of patients attending primary care clinics (not measured by prevalence estimates). Of particular interest is the subgroup of patients with DBMA10+ in each sample. The mean DBMA score for patients in the DBMA10+ subgroup was significantly higher in those attending primary care clinics (Table 1). The age-standardized prevalence was almost double in the same group as compared with the sample from the general population (Table 3). It is very likely that we may find among this group the most complex patients with an important level of health care utilization and higher costs for the health care system [10, 11].

Our study has limitations. Regarding the measurement of multimorbidity, we considered 21 frequent chronic conditions, and this has an influence on prevalence estimates of multimorbidity. We have previously reported that a longer list of conditions would result in higher prevalence estimates [2]. Also, we had to rely on the self-reported presence of chronic conditions to measure multimorbidity and, hence, either over-reporting or under-reporting may have occurred [12, 13]. Finally, caution should be taken when the results of this study are extrapolated to different contexts or to other populations different from the Canadian population where the study was conducted.

## Conclusions

We found that there is a difference of about 10 % in prevalence estimates of multimorbidity between samples from the general population and primary care clinical practices which is not affected by the use of different operational definitions of multimorbidity. The study suggests that the problem of multimorbidity in the two settings is different both quantitatively (a higher proportion of patients with multimorbidity in primary care clinical practices), and qualitatively (a higher disease burden of patients attending primary care clinics). For decision-makers interested in resource allocation, prevalence estimates in samples from primary care practices are more informative than estimates in the general population but burden of disease should also be considered as it results in more complexity in primary care clinical practices.

## Appendix

See Table 4.

**Table 4:** Prevalence estimates by sex without adjustment in samples from the general population and primary care practices according to different operational definitions of multimorbidity (MM)

	General population % (n with MM/total sample)		p value*	Primary care % (n with MM/total sample)		p value
	Males	Females		Males	Females	
MM 2+	59.5 (416/699)	59.3 (604/1019)	0.921	70.4 (178/253)	69.0 (370/536)	0.706
MM 3+	42.5 (297/699)	44.6 (454/1019)	0.397	53.8 (136/253)	54.7 (293/536)	0.811
DBMA 10+	14.9 (104/699)	18.4 (187/1019)	0.059	28.5 (72/253)	25.6 (137/536)	0.389

*MM 2+* subjects with two or more chronic conditions; *MM 3+* subjects with three or more chronic conditions; *DBMA 10+* subjects with DBMA score of 10 or higher

* Chi square

## Abbreviations

MM 2+: subjects with two or more chronic conditions
MM 3+: subjects with three or more chronic conditions
DBMA: disease burden morbidity assessment
DBMA 10+: subjects with DBMA score of 10 or higher
